# Mass‐Forming NSAID‐Associated Colopathy Mimicking Right‐Sided Colon Cancer: A Case Report

**DOI:** 10.1155/crip/2605428

**Published:** 2026-08-19

**Authors:** Hong Yu, Sonalben L. Italiya, Hongbo Wang, Jason Koshy, Ya Xu

**Affiliations:** ^1^ Department of Pathology and Immunology, Baylor College of Medicine, Houston, Texas, USA, bcm.edu; ^2^ Department of Surgery, Pathology Service, HCA Houston Healthcare Clear Lake, Webster, Texas, USA

**Keywords:** aspirin, colon cancer mimic, mass-forming colonic lesion, NSAID-associated colopathy

## Abstract

Nonsteroidal anti‐inflammatory drugs (NSAIDs) can cause mucosal injury throughout the gastrointestinal tract, yet NSAID‐related colonic damage remains underrecognized. We report a rare case of an ulcerated colonic mass mimicking carcinoma in a patient with long‐term NSAID exposure. The patient, a 63‐year‐old man with a history of coronary artery disease and coronary artery bypass grafting more than 25 years earlier, was receiving long‐term dual antiplatelet therapy consisting of aspirin (81 mg daily) and clopidogrel (75 mg daily), as well as chronic pantoprazole therapy. He presented with a 4‐day history of hematochezia without weight loss. Esophagogastroduodenoscopy revealed no bleeding source, but colonoscopy identified an ulcerated, nonobstructive mass in the cecum and proximal ascending colon. A biopsy revealed colonic mucosa with severe ulceration and granulation tissue. The subsequent right hemicolectomy specimen revealed a 5.5‐cm, ill‐defined ulcerative mass in the cecum and ascending colon. Microscopically, the cecum and ascending colon showed ulcerated mucosa with fibrosis, reactive changes, and foreign body material deposition, without evidence of malignancy. Immunohistochemical stains for cytomegalovirus (CMV) and herpes simplex virus (HSV) were negative. Following surgery, aspirin and clopidogrel were resumed because of the patient′s cardiovascular risk. Approximately 1 year later, his hemoglobin level was 12.0 g/dL (6.7 g/dL before surgery), and no gastrointestinal bleeding, diarrhea, or abdominal pain was documented during that encounter. Because aspirin was continued, a clinical response to medication withdrawal could not be assessed. Based on the medication history, right‐sided distribution, and histologic findings, the lesion was considered most consistent with suspected aspirin‐associated colopathy presenting as a mass‐like lesion. However, the concomitant clopidogrel and pantoprazole use and the absence of a formal aspirin dechallenge limited definitive causal attribution.

## 1. Introduction

Nonsteroidal anti‐inflammatory drugs (NSAIDs) are widely used agents capable of causing mucosal injury throughout the gastrointestinal tract [1, 2]. While upper gastrointestinal toxicity is well recognized, NSAID‐induced colonic injury is comparatively underappreciated [3, 4]. The right colon, particularly the cecum and proximal ascending colon, is most frequently involved [3–5]. Although NSAID‐induced ulcers typically present as sharply demarcated mucosal defects, a minority evolve into mass‐forming lesions that closely resemble malignancy on endoscopy or imaging [5, 6].

The true incidence of NSAID‐associated colopathy remains unknown because population‐based studies are lacking and the condition is likely underrecognized owing to its nonspecific clinical and endoscopic manifestations. Colonic ulceration is the most commonly described form of NSAID‐associated colonic injury, whereas diaphragm‐like strictures and mass‐forming lesions are uncommon. A 2013 systematic review identified 45 reported cases of NSAID‐associated colonic diaphragm disease, most commonly affecting patients in the seventh decade, with a marked female predominance and preferential involvement of the ascending colon [3, 7]. A more recent 2025 literature review expanded the number of reported cases of colonic diaphragm disease to 53 [8]. Although nonmass‐forming colonic ulcers are a recognized manifestation of NSAID‐associated injury, the specific mass‐forming ulcerative phenotype that mimics carcinoma remains exceptionally rare and is described primarily in isolated case reports and small case series [4]. A recent 2025 case report described a synchronous aspirin‐induced ileal ulcer initially suspected to be malignant alongside an ascending colon adenocarcinoma, further illustrating the diagnostic challenges posed by aspirin‐associated mucosal injury [9].

The mechanisms underlying NSAID‐induced colonic injury involve both cyclooxygenase‐dependent prostaglandin depletion, which reduces mucosal blood flow and barrier integrity, and cyclooxygenase‐independent mitochondrial injury, including uncoupling of oxidative phosphorylation and increased epithelial permeability [10–12]. Collectively, these processes predispose the mucosa to ischemic‐type injury and reparative fibroinflammatory responses.

Endoscopic findings range from discrete ulcers to semilunar or circumferential lesions and, less commonly, the diaphragm‐like strictures characteristic of diaphragm disease [7]. When mass formation occurs, differentiation from neoplasia often requires histologic evaluation. We report a rare case of suspected aspirin‐associated colopathy presenting as a large, ulcerated mass in the cecum and proximal ascending colon and highlight the clinicopathologic features and causality considerations essential for accurate diagnosis.

## 2. Case Presentation

A 63‐year‐old man with a history of coronary artery disease and coronary artery bypass grafting more than 25 years earlier presented with a 4‐day history of hematochezia. Laboratory evaluation showed severe anemia, with a hemoglobin level of 6.7 g/dL, and fecal occult blood testing was positive. C‐reactive protein (CRP) and erythrocyte sedimentation rate (ESR) results were not available in the medical record. No infectious stool‐testing results, including bacterial culture, *Clostridioides difficile* testing, or ova and parasite examination, were available. The patient did not report diarrhea, fever, abdominal pain, weight loss, or constitutional symptoms. His medication history was notable for long‐term dual antiplatelet therapy with aspirin (81 mg daily) and clopidogrel (75 mg daily), as well as chronic pantoprazole therapy. He was not taking nonaspirin NSAIDs, systemic anticoagulants, biologic agents, or other medications commonly associated with intestinal ulceration. Pantoprazole was continued intravenously during hospitalization.

Computed tomography (CT) of the abdomen and pelvis showed no bowel obstruction or active contrast extravasation and only minimal colonic diverticulosis. The CT report did not identify a discrete cecal or ascending colon mass. Esophagogastroduodenoscopy showed no source of bleeding. Colonoscopy demonstrated an ulcerated, nonobstructive mass involving the cecum and proximal ascending colon, highly suspicious for right‐sided colon cancer. Thus, the concern for malignancy was based primarily on the endoscopic appearance rather than the CT findings. Endoscopic biopsies showed severe ulceration and granulation tissue, without dysplasia or carcinoma. Because the biopsy predominantly sampled the ulcer bed, the absence of malignant cells did not reliably exclude an unsampled underlying neoplasm. In the setting of gastrointestinal bleeding, severe anemia, and a large endoscopically concerning mass, surgical management was pursued, and a right hemicolectomy was performed. No repeat endoscopic biopsy was documented before surgery. Gross examination revealed a 5.5‐cm, ill‐defined ulcerated mass.

**Figure 1:**
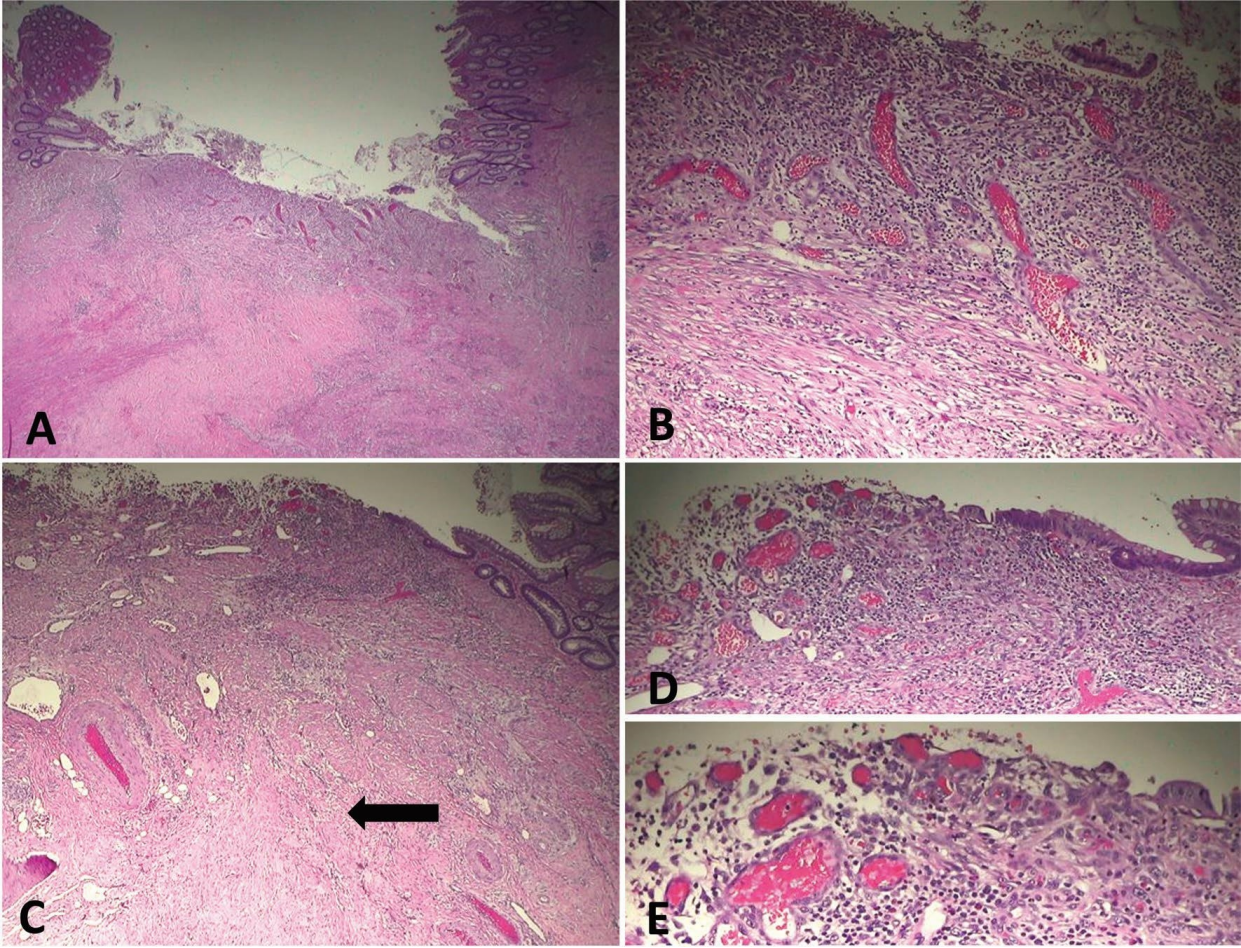
Histologic sections of the right hemicolectomy specimen show an ulcerative mass involving the cecum and ascending colon (A, 20×), without atypical epithelial cells in the ulcer bed (B, 100×). The ulcer shows thickening of the muscularis mucosae and extensive submucosal fibrosis (arrow) with a relatively sharp transition to preserved adjacent mucosa (C, 20×). The inflamed mucosa shows reactive epithelial changes (D, 100×) and a mixed lamina propria inflammatory infiltrate (E, 200×).

**Figure 2:**
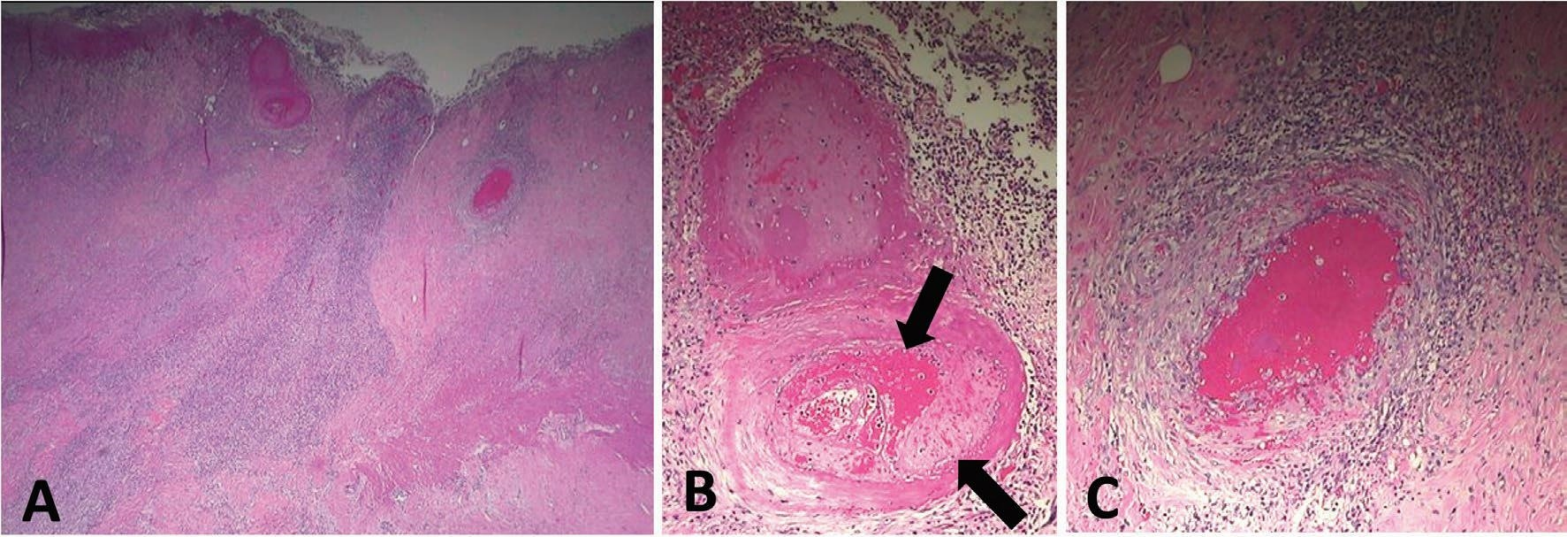
Medium‐sized submucosal vessels within the ulcer bed show vascular injury (A, 20×), including intimal thickening and luminal fibrin deposition (arrow) (B, 100×), and focal mixed mural inflammation in continuity with the surrounding ulcer‐associated inflammatory process (C, 100×). In the absence of fibrinoid necrosis or leukocytoclastic change, these findings were interpreted as localized secondary vascular injury rather than primary vasculitis.

**Figure 3:**
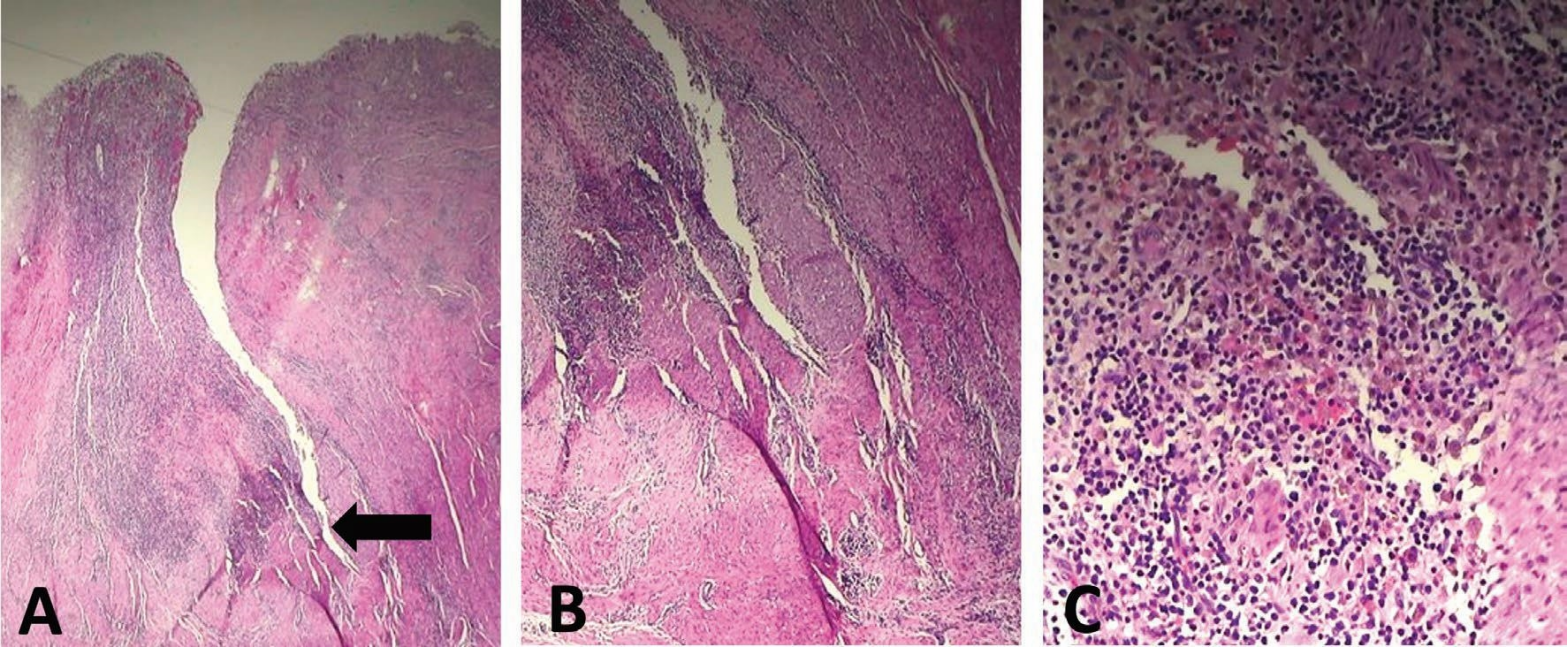
Focal fissure‐like ulceration extends toward the muscularis propria (arrow) (A, 20×; B, 40×). The fissure contains predominantly acute neutrophilic inflammation (C, 200×), without granulomatous inflammation or other convincing features of Crohn′s disease.

**Figure 4:**
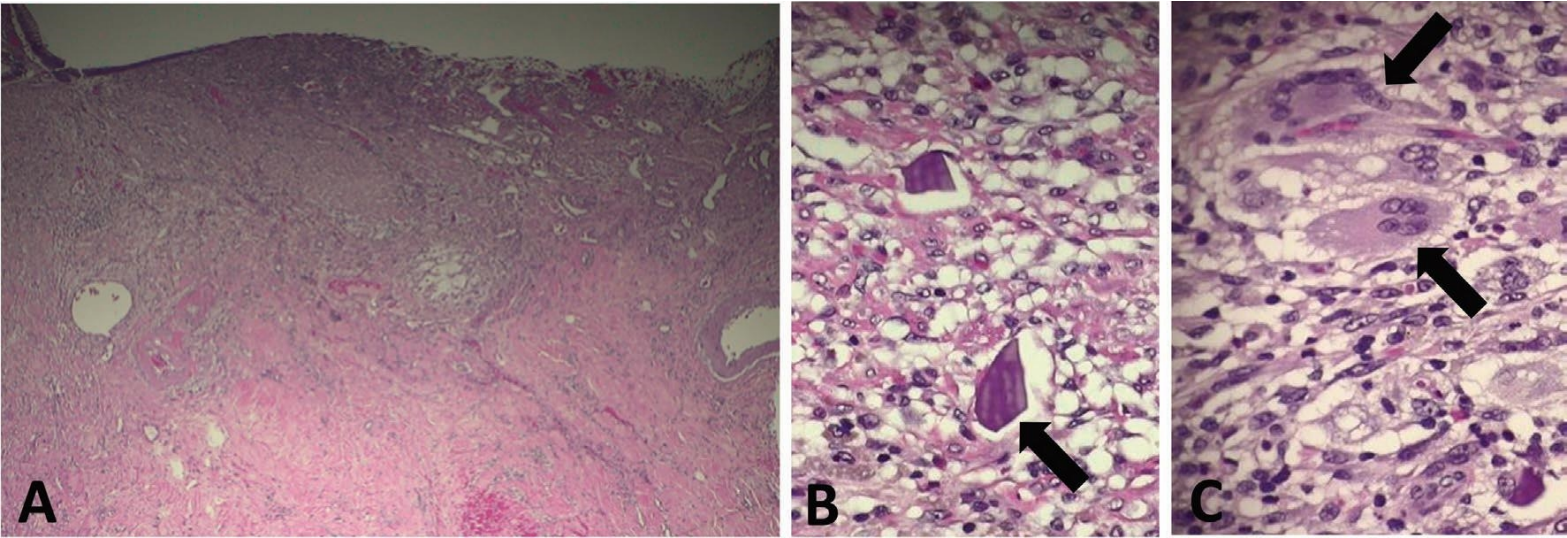
Foreign body deposition (arrow) and an associated multinucleated giant cell reaction (arrow) are present in the submucosa of the ulcer (A, 40×). Higher power views show bipyramidal or envelope‐shaped purple foreign material (arrow) (B, 400×) and multinucleated giant cells (arrow) (C, 400×).

**Figure 5:**
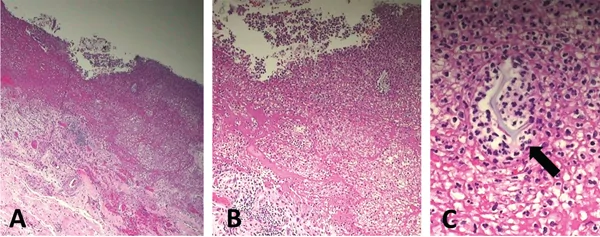
The ulcerated lesion shows focal ongoing activity (A, 40×), including acute inflammatory exudate (B, 100×) and luminal food material (arrow) (C, 400×).

**Figure 6:**
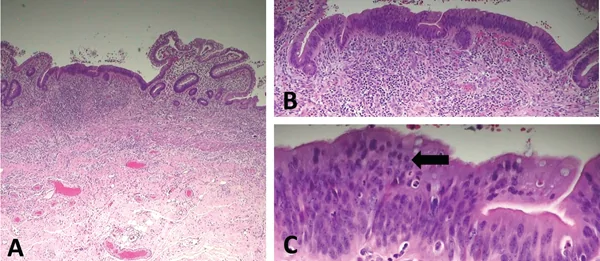
The mucosa adjacent to the ulcer shows localized architectural distortion (A, 40×), mixed lamina propria inflammation, and reactive epithelium (B, 100×). Focal epithelial pseudostratification resembling a tubular adenoma is highlighted by the arrow (C, 400×).

**Figure 7:**
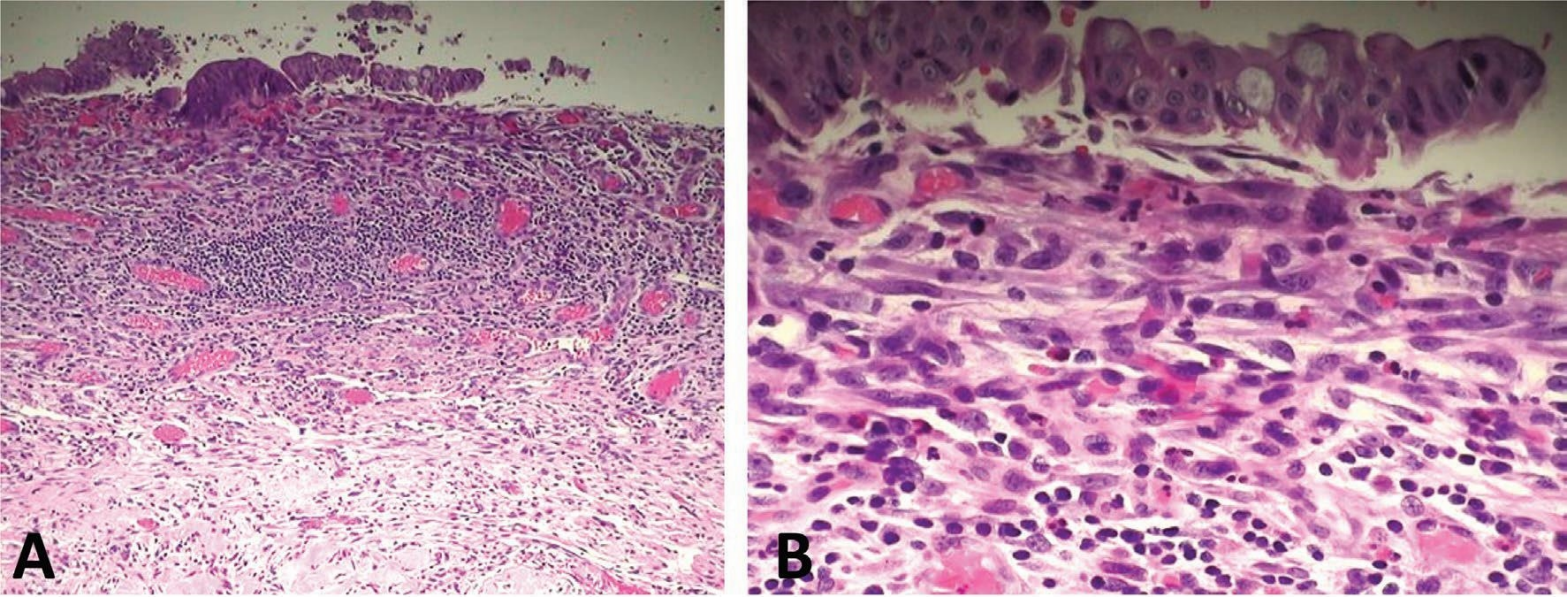
The mucosa adjacent to the ulcer shows a single layer of reparative/regenerating epithelial cells (A, 100×). A high‐power view demonstrates re‐epithelializing cells with rare goblet cells overlying granulation‐type stroma (B, 400×).

Microscopically, the lesion showed ulcerated colonic mucosa with lamina propria hyalinization (Figure 1A,B), granulation tissue, submucosal fibrosis, and reactive epithelial changes (Figure 1C–E). Medium‐sized submucosal vessels within and adjacent to the ulcer bed showed localized vascular injury characterized by intimal thickening, luminal fibrin deposition, and mixed mural inflammation (Figure 2A–C). These changes were predominantly confined to the involved bowel segment and were interpreted as secondary vascular injury associated with the ulcerative process rather than evidence of a primary systemic vasculitis. The available clinical record documented no systemic manifestations suggestive of a primary vasculitic disorder; however, a dedicated rheumatologic evaluation, including serologic testing, was not performed. Deeper sections showed fissure‐like ulceration extending toward the muscularis propria (Figure 3A–C). Although this finding raised consideration of Crohn′s disease, the fissuring was localized to the ulcerated mass and was not accompanied by granulomas, transmural lymphoid aggregates, or diffuse chronic mucosal injury. Foreign‐body material deposition with a foreign‐body–type multinucleated giant cell reaction was present within the ulcer base (Figure 4A–C). Areas of ongoing acute inflammatory activity, including neutrophilic exudate and luminal food material, were also identified (Figure 5A–C). The mucosa adjacent to the ulcer demonstrated architectural distortion and reactive epithelial changes, including focal pseudostratification resembling a tubular adenoma (Figure 6A–C), whereas other regions showed reparative single‐layer epithelium and granulation‐type stroma (Figure 7A,B).

No dysplasia or invasive carcinoma was identified. Cytomegalovirus (CMV) and herpes simplex virus (HSV) immunostains were negative. In the context of long‐term low‐dose aspirin exposure, right‐sided disease distribution, and the histologic findings, the lesion was considered most consistent with suspected aspirin‐associated colopathy. However, this interpretation remained a clinicopathologic diagnosis of exclusion. Following consultation with cardiology, aspirin and clopidogrel were resumed postoperatively because of the patient′s substantial cardiovascular risk. Approximately 1 year later, his hemoglobin had increased to 12.0 g/dL, and no recurrent gastrointestinal bleeding, diarrhea, or abdominal pain was documented during that encounter.

## 3. Discussion

NSAID‐related injury of the lower gastrointestinal tract remains underrecognized, despite longstanding evidence that chronic NSAID exposure can damage both the small and large intestine and present with bleeding, anemia, abdominal pain, or iron‐deficiency states [1, 2, 13]. The right colon, particularly the cecum and proximal ascending colon, is disproportionately affected, likely due to higher luminal drug concentrations, enterohepatic recirculation, and increased mucosal vulnerability from prostaglandin depletion and mitochondrial toxicity [14]. Through both cyclooxygenase‐dependent and cyclooxygenase‐independent mechanisms, NSAIDs reduce mucosal blood flow, impair barrier integrity, and increase epithelial permeability, rendering the mucosa vulnerable to ischemic‐type injury and fibrotic repair [10–12].

Endoscopic and gross appearances range from sharply demarcated ulcers to semilunar or circumferential defects, as well as the classic diaphragm‐like strictures characteristic of NSAID‐related diaphragm disease [4, 7]. Much less frequently, chronic injury from NSAIDs results in an ulcerated mass‐forming lesion that mimics carcinoma clinically, radiographically, and endoscopically, an important diagnostic pitfall that may necessitate surgical resection when malignancy cannot be confidently excluded by endoscopic biopsy [8, 12]. In this case, long‐term low‐dose aspirin exposure was considered a possible contributor to the development of the large, ulcerated mass in the cecum and proximal ascending colon, which was initially indistinguishable from right‐sided colon cancer. Nevertheless, a definitive causal relationship could not be established.

The patient was also receiving clopidogrel as part of dual antiplatelet therapy and was taking pantoprazole. Clopidogrel may have increased the severity of bleeding from the ulcerated lesion, although its role in producing the characteristic localized ulceration and submucosal fibrosis of NSAID‐associated colopathy is less established [15–17]. Although proton pump inhibitors reduce upper gastrointestinal injury, they do not reliably protect the lower gastrointestinal tract and may modify aspirin‐ or NSAID‐associated intestinal injury; however, their role in mass‐forming colonic injury remains uncertain [18, 19]. No exposure to nonaspirin NSAIDs, systemic anticoagulants, or biologic agents was identified. Because aspirin was resumed after surgery and no formal dechallenge or rechallenge assessment was performed, attribution to aspirin remains a clinicopathologic diagnosis of exclusion. We therefore classify the lesion as suspected aspirin‐associated colopathy rather than definitively aspirin‐induced colopathy.

Histopathology is decisive when interpreted alongside medication history [3, 20]. NSAID‐induced colopathy frequently shows features overlapping with ischemic injury, including mucosal ulceration or necrosis, lamina propria hyalinization, granulation tissue, submucosal fibrosis, and variable reactive epithelial changes [4, 21, 22]. Foreign‐body material accompanied by a foreign‐body–type giant cell reaction was also present within the ulcer bed. Because this finding is nonspecific and may occur as part of an ulcer‐associated inflammatory or reparative response, it was not considered diagnostic of NSAID‐associated injury. Importantly, NSAID‐induced lesions lack cytologic dysplasia, infiltrative glands, or desmoplastic stromal response [20]. When biopsy samples are superficial and dominated by ulcer bed and granulation tissue, reactive atypia can raise concern for malignancy; in such circumstances, careful correlation with clinical history, imaging findings, and endoscopic appearance is essential to avoid misclassification [6, 23]. Infectious mimics, especially CMV in elderly or immunosuppressed patients, should also be excluded; in this case, negative CMV and HSV immunostains supported a noninfectious medication‐related injury [24, 25].

The differential diagnosis included Crohn′s disease, ischemic colitis, primary or drug‐induced vasculitis, infectious colitis, and segmental colitis associated with diverticulosis (SCAD), in addition to adenocarcinoma [13, 26]. Crohn′s disease was considered because of the focal fissure‐like ulceration. However, the lesion was localized to the cecum and proximal ascending colon and lacked noncrypt injury‐associated granulomas, transmural lymphoid aggregates, and diffuse or multifocal chronic mucosal injury [3, 27]. Although focal crypt architectural distortion was present adjacent to the ulcer, it occurred in a reparative setting and was not accompanied by diffuse basal plasmacytosis or widespread chronic architectural abnormalities [28]. Thus, the fissure‐like ulceration alone was insufficient to support Crohn′s disease [26, 29].

Ischemic colitis remained an important morphologic consideration because lamina propria hyalinization, ulceration, fibrosis, and vascular injury overlap with an ischemic pattern, and mass‐forming ischemic colitis can rarely involve the right colon [22, 30, 31]. The patient′s cardiovascular disease also represented a potential ischemic risk factor. Nevertheless, no discrete thromboembolic or occlusive vascular lesion was identified in the resection specimen, and the localized right‐sided injury in the setting of long‐term aspirin exposure favored medication‐associated injury [13, 21]. Because the histologic overlap is substantial, an ischemic contribution cannot be entirely excluded.

Primary systemic or drug‐induced vasculitis was also considered because of mural inflammation and fibrin deposition in submucosal vessels [26, 32]. However, the vascular changes were localized to the ulcer bed and were not accompanied by fibrinoid necrosis, leukocytoclasia, granulomatous vascular inflammation, or vasculitis in uninvolved bowel. The available clinical record documented no systemic manifestations suggesting a primary vasculitic disorder. These findings favored secondary vascular injury within an active ulcer rather than primary systemic vasculitis [32].

An infectious etiology was not favored because no viral cytopathic change was identified, CMV and HSV immunohistochemical stains were negative, and the patient was not receiving biologic or other immunosuppressive therapy. Although these findings cannot exclude every viral infection, the absence of cytopathic change or a compatible clinical syndrome made an untested viral etiology unlikely. Finally, SCAD was considered but was not supported because only minimal diverticulosis was identified radiographically, whereas the inflammatory mass involved the cecum and proximal ascending colon rather than a diverticula‐bearing sigmoid segment [33, 34]. Collectively, the distribution, medication exposure, and pathologic findings favored suspected aspirin‐associated colopathy, while acknowledging morphologic overlap with ischemic injury.

The vascular findings warrant specific clarification because overt vasculitis is not a characteristic diagnostic feature of NSAID‐associated colopathy [32, 35]. In this case, mural inflammation, intimal thickening, and luminal fibrin deposition were identified in vessels within the ulcer bed. On review, the vascular inflammation was localized to the ulcerated region and occurred in continuity with the surrounding inflammatory process. There was no convincing fibrinoid necrosis, leukocytoclasia, granulomatous vascular inflammation, or similar vascular injury in uninvolved bowel [36]. The vascular changes were therefore interpreted as secondary to the local ulcerative process rather than as a key diagnostic feature of aspirin‐associated colopathy or evidence of primary systemic vasculitis [32, 37].

Management of suspected NSAID‐associated colopathy depends on the patient′s clinical stability, severity of bleeding, degree of diagnostic certainty, and presence of structural complications [38–40]. When the patient is stable and malignancy is considered unlikely, withdrawal of the suspected medication followed by short‐interval repeat endoscopy and biopsy may demonstrate regression and help avoid surgery. Surgery may nevertheless be warranted in patients with ongoing or severe bleeding, obstruction or perforation, or a mass‐like lesion for which malignancy cannot be reliably excluded despite endoscopic sampling.

In the present case, the initial biopsy showed only ulceration and granulation tissue. Although no malignant cells were identified, the biopsy was superficial and did not definitely exclude an underlying neoplasm. The patient also had marked anemia, with a hemoglobin level of 6.7 g/dL, and a large, ulcerated right‐colon mass that was considered highly suspicious for carcinoma endoscopically. CT showed no obstruction or active contrast extravasation. Given the severity of the clinical presentation and the persistent concern for an unsampled malignancy, right hemicolectomy was performed. A repeat biopsy or trial of aspirin withdrawal was not undertaken before surgery. Final examination of the resection specimen excluded malignancy and established the benign ulcerative and fibrosing nature of the lesion. Aspirin and clopidogrel were subsequently resumed because of the patient′s substantial cardiovascular risk.

In summary, this case illustrates a mass‐forming colonic lesion suspected to be associated with long‐term low‐dose aspirin exposure, an uncommon but clinically consequential mimic of colon cancer. Three practice points emerge: (1) obtain detailed NSAID exposure history in patients with right‐sided ulcerative or mass‐like lesions; (2) recognize a histologic pattern compatible with medication‐associated injury, including ulceration with reparative fibrosis and the absence of dysplasia or invasive carcinoma; and (3) when the patient is clinically stable and malignancy is considered sufficiently unlikely, consider medication withdrawal with short‐interval endoscopic reassessment before colectomy. Increased awareness of this entity can reduce diagnostic uncertainty and prevent overtreatment while ensuring appropriate intervention when necessary.

## 4. Conclusions

NSAID‐associated colonic ulceration can, in rare circumstances, manifest as a mass‐forming lesion that is virtually indistinguishable from carcinoma before histologic assessment. In this patient, the medication history, right‐sided distribution, and ulceration with reparative fibrosis supported the diagnosis of suspected aspirin‐associated colopathy after exclusion of malignancy and other major competing etiologies. However, concomitant clopidogrel and pantoprazole use and the absence of aspirin withdrawal limited definitive causal attribution. Careful integration of medication exposure, endoscopic findings, histopathologic features, and competing etiologies is therefore essential. When clinically appropriate, withdrawal of the suspected medication with short‐interval reassessment may help clarify the diagnosis and guide the decision between continued endoscopic evaluation and surgical intervention.

## Funding

No funding was received for this manuscript.

## Conflicts of Interest

The authors declare no conflicts of interest.

## Data Availability

The data that support the findings of this study are available from the corresponding author upon reasonable request.
